# Copper-Nanocoated Ultra-Small Cells in Grain Boundaries Inside an Extinct Vent Chimney

**DOI:** 10.3389/fmicb.2022.864205

**Published:** 2022-06-07

**Authors:** Hinako Takamiya, Mariko Kouduka, Hitoshi Furutani, Hiroki Mukai, Kaoru Nakagawa, Takushi Yamamoto, Shingo Kato, Yu Kodama, Naotaka Tomioka, Motoo Ito, Yohey Suzuki

**Affiliations:** ^1^Department of Earth and Planetary Science, The University of Tokyo, Bunkyo City, Japan; ^2^Faculty of Life and Environmental Sciences, University of Tsukuba, Tsukuba, Japan; ^3^Solutions COE, Analytical & Measuring Instruments Division, Shimadzu Corporation, Kyoto, Japan; ^4^Japan Collection of Microorganisms (JCM), RIKEN BioResource Research Center, Tsukuba, Japan; ^5^TOYO Corporation, Chuo City, Japan; ^6^Kochi Institute for Core Sample Research, Japan Agency for Marine-Earth Science and Technology (JAMSTEC), Nankoku, Japan

**Keywords:** metal sulfide deposits, submicron-scale biosignature analyses, nanometer-scale solid characterizations, rock-hosted life, deep-sea hydrothermal vent

## Abstract

Chemosynthetic organisms flourish around deep-sea hydrothermal vents where energy-rich fluids are emitted from metal sulfide chimneys. However, microbial life hosted in mineral assemblages in extinct chimneys lacking fluid venting remains largely unknown. The interior of extinct chimneys remains anoxic where the percolation of oxygenated seawater is limited within tightly packed metal sulfide grains. Given the scarcity of photosynthetic organics in deep seawater, anaerobic microbes might inhabit the grain boundaries energetically depending on substrates derived from rock-water interactions. In this study, we reported ultra-small cells directly visualized in grain boundaries of CuFeS_2_ inside an extinct metal sulfide chimney from the southern Mariana Trough. Nanoscale solid analyses reveal that ultra-small cells are coated with Cu_2_O nanocrystals in grain boundaries enriched with C, N, and P. *In situ* spectroscopic and spectrometric characterizations demonstrate the distribution of organics with amide groups and a large molecular organic compound in the grain boundaries. We inferred that the ultra-small cells are anaerobes because of the fast dissolution of Cu_2_O nanocrystals in oxygenated solution. This Cu_2_O property also excludes the possibility of microbial contamination from ambient seawater during sampling. It is shown by 16S rRNA gene sequence analysis that the chimney interior is dominated by Pacearchaeota known to have anaerobic metabolisms and ultra-small cells. Our results support the potential existence of photosynthesis-independent microbial ecosystems in grain boundaries in submarine metal sulfides deposits on the early Earth.

## Introduction

Deep-sea hydrothermal fluid venting by “black smokers” is associated with the extensive eruption of pillow lava on the deep seafloor (Corliss et al., 1979; Elderfield and Schultz, 1996). Seawater is recharged, interacts with extrusive and intrusive basaltic rocks, and is then discharged as high-temperature hydrothermal fluid enriched with heavy metals and chemicals that can be used for microbial energy generation, such as HS^–^, Fe(II), CH_4_, and H_2_ (Martin et al., 2008). As a result of rapid cooling of hydrothermal fluid, vent chimneys tend to form with an internal zonation of mineral assemblages (Schulz and Zabel, 2006). Decreasing temperature and increasing pH cause the sequential deposition of minerals. In general, chalcopyrite (CuFeS_2_) precipitated from high-temperatures fluid (>300°C) tends to form the inner wall with tight packing of chalcopyrite grains, which is surrounded by marcasite (FeS_2_), pyrite (FeS_2_), and sphalerite (ZnS) precipitated from lower temperatures in outer porous layers.

Around black smoker chimneys, the dark oasis densely colonized by peculiar organisms such as tubeworms and giant clams is thought to be dependent on chemicals emitted from the geothermally sourced fluid (Felbeck, 1981). Microbial populations thriving in actively venting chimneys have been studied intensively (Dick, 2019 and references therein). In addition to active chimneys with excess energy supplies from hydrothermal fluids, extinct chimneys without fluid venting appear to host chemolithotrophic microbes with scarce energy supplies available solely from metal sulfide minerals. Dominant microbial populations are similar in geographically and mineralogically distinct chimneys (Suzuki et al., 2004a; Kato et al., 2010; Sylvan et al., 2012, 2013; Han et al., 2018). However, their proportion in microbial composition tends to vary significantly, according to the differences in porosity, permeability, and mineral assemblage (Toner et al., 2013). To understand the ecological features of microbial populations, it is critical to spatially correlate the distributions of microbial cells to the chimney structure. In this study, we observed the inside of an extinct vent chimney to determine the distributions of microbial cells and their association with metal sulfide minerals. In addition, we performed 16S rRNA gene sequence analysis to clarify microbial populations in the extinct vent chimney.

## Results

### Extinct Chimney Inner Wall With Microbial Signals

**FIGURE 1:**
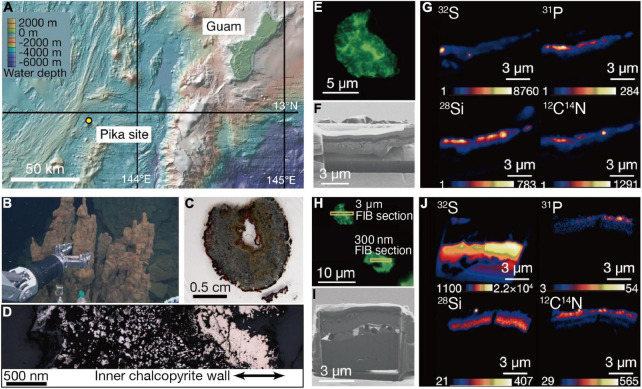
Sampling of an extinct chimney and microbial signal detections. **(A)** Bathymetric map of the southern Marina trough showing the Pika site and Guam. **(B)** Photograph showing the extinct chimney sampled by the remotely operated vehicle *Hyper Dolphin*. **(C)** Light microscopy image of a thin section from the extinct chimney. **(D)** Reflection light microscope image of the thin section. The inner chalcopyrite wall is indicated with arrows. **(E)** Fluorescence microscopy image of a grain boundary with greenish cell-like signals where a 3-μm thick focused ion beam (FIB) section was fabricated. **(F)** Ga ion image of a 3-μm thick FIB section from the grain boundary shown in **(E)**. **(G)** Nanoscale secondary ion mass spectrometry (NanoSIMS) images of the FIB-fabricated grain boundary with intensity color contours. **(H)** Fluorescence microscopy image of a grain boundary with greenish cell-like signals where a 300-nm thick FIB section was fabricated. **(I)** Ga ion image of a 300-nm thick FIB section as pointed in **(H)**. **(J)** NanoSIMS images of the FIB-fabricated grain boundary with intensity color contours.

The Pika site is a recently discovered deep-sea hydrothermal field with black smokers (>300°C) in the southern Mariana Trough, about 140 km east of Guam (Figure 1A; Nakamura et al., 2013). Chimney samples were collected by the remotely operated vehicle Hyper-Dolphin at a water depth and temperature of 2,787 m and 1.7°C, respectively (Figure 1B). Zonation characteristic of metal sulfide chimneys was found in a thin section of one of the chimney samples (Figure 1C). There was an unaltered gold-colored part on the inner wall of chalcopyrite directly deposited from black smokers (Figure 1D).

Analytical procedures have been developed to visualize and quantify microbial cells hosted in crack-infilling minerals in the oceanic crust by preparing thin sections of rocks embedded in hydrophilic resin called LR White (Sueoka et al., 2019; Suzuki et al., 2020). Microbial cells embedded in the resin are stainable with a DNA dye called SYBR-Green I. Microscopic examinations of the inner wall of the chimney prepared as described above revealed the presence of patches with cell-like fluorescent signals where visible light was not transmitted (Supplementary Figure 1). Focused ion beam (FIB) sections with thicknesses of 3 μm (Figures 1E–G) and 300 nm (Figures 1H–J) were fabricated to characterize patches associated with the cell-like signals by nanoscale secondary ion mass spectrometry (NanoSIMS). In the 3-μm thick FIB section, signals from ^32^S, ^12^C^14^N, and ^31^P were detected in a silica-bearing layer with sub-micron voids (Figure 1G and Supplementary Figure 2). Similar results were obtained from the 300-nm thick FIB section (Figure 1J). A selected area electron diffraction (SAED) pattern showed that the silica-bearing layer also observed by transmission electron microscopy (TEM) was composed of amorphous material (Supplementary Figure 3).

### Microbial Cells Visualized in Chalcopyrite Grain Boundaries

**FIGURE 2:**
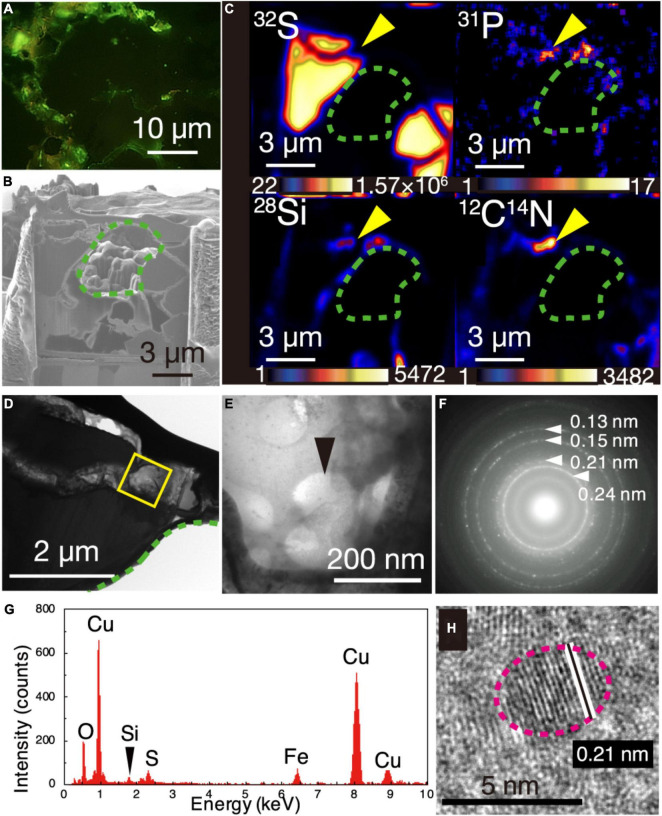
Visualization of microbial cells associated with Cu-bearing nanocrystals in chalcopyrite grain boundaries. **(A)** Fluorescence microscopy image of a grain boundary with greenish cell-like signals where a 150-nm thick FIB section was fabricated. **(B)** Ga ion image of a 150-nm thick FIB section of the grain boundary shown in **(A)**. A green dotted line shows a hole corresponding to chalcopyrite grains in the FIB section. **(C)** NanoSIMS images of the 150-nm thick FIB section with intensity color contours. Yellow arrows indicate the region presented in **(E)**. **(D)** TEM image of a ribbon-shaped grain boundary of a 150-nm thick FIB section. A yellow square indicates the same region presented in **(E)**. **(E)** TEM image of small spheres. **(F)** Selected area electron diffraction pattern from the small spheres in **(E)**. Arrows indicate ring patterns with interplanar spacings. **(G)** Energy dispersive X-ray spectrum from the small spheres. **(H)** High-resolution TEM image of crystalline nanoparticles in the region pointed by a black arrow in **(E)**. White lines indicate lattice fringes of a nanocrystal with the rim indicated by a pink dotted line.

The greenish cell-like signals from the DNA dye and the overlapped NanoSIMS mapping of P and CN are consistent with results from a previous study of microbial cells in mineral-filled cracks (Suzuki et al., 2020). However, the appearance of individual cells was not clear, in contrast to the earlier work. To clearly observe individual cells, a 150-nm thick FIB section was fabricated from a chalcopyrite grain boundary associated with silica (Supplementary Figure 4) where greenish cell-like signals without light transmission were observed after SYBR-Green I staining (Figure 2A). During the FIB fabrication, some chalcopyrite grains collapsed, leaving a hole in the section where chalcopyrite grains were surrounded by grain boundaries with width <1 μm (Figure 2B). It was revealed by NanoSIMS analysis that ^12^C^14^N, ^28^Si, and ^31^P were overlapped in a ribbon-shaped grain boundary (Figures 2B,C). TEM observations of the region with overlapping ^31^P, ^12^C^14^N, and ^28^Si showed small spheres with diameters of < 200 nm (Figures 2D,E). Cuprite (Cu_2_O) was identified by a SAED pattern (Figure 2F) and an energy-dispersive X-ray spectrum (EDS) (Figure 2G). High-resolution TEM observations revealed that ∼5-nm diameter particles of cuprite were spatially associated with the small spheres (Figure 2H).

**FIGURE 3:**
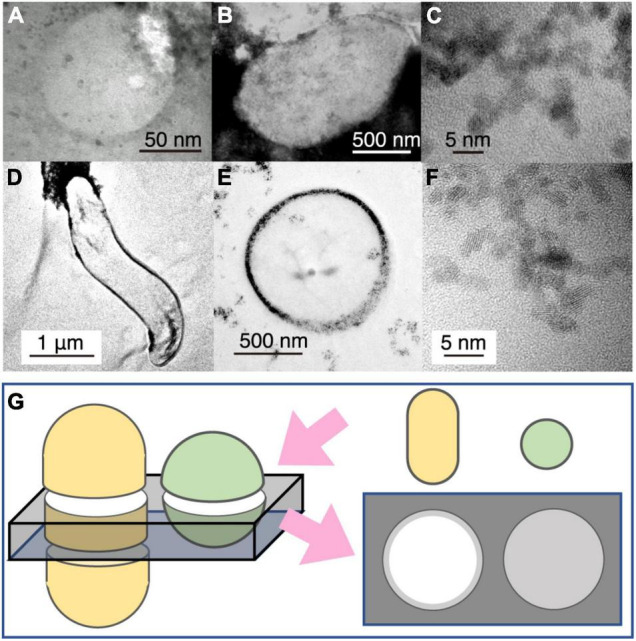
Transmission electron microscopy (TEM) image contrast from microbial cells associated with extracellular nanoparticles. **(A)** Typical small sphere observed in Figure 2E. **(B)** TEM image of *Geobacter sulfureducens* extracellularly precipitated with uraninite nanoparticles. **(C)** High-resolution TEM image of uraninite nanoparticles produced by *G. sulfureducens.* **(D,E)** TEM image of *Desulfovibrio desulfuricans* with periplasmic uraninite nanoparticles. **(F)**, High-resolution TEM image of uraninite nanoparticles produced by *D. desulfruicans.* **(G)** Schematic explanation of the appearance of microbial cells extracellularly coated with nanoparticles by TEM observations. This illustration shows how a 150-nm thick FIB section was fabricated for rod and coccoid cells (left) and an expected TEM image from the FIB section (right). Gray color indicates the presence of extracellular nanoparticles in the right illustration.

To help interpret the TEM image of the small spheres associated with cuprite nanoparticles (Figures 2E, 3A), TEM observations were performed for cultured cells of *Geobacter sulfurreducens* associated with extracellularly precipitated nanocrystals of uraninite (UO_2_) (Figures 3B,C) and those of *Desulfovibrio desulfuricans* with UO_2_ nanoparticles in the periplasmic space (Figures 3D–F). UO_2_ nanoparticles were used for understanding the effects of nanoparticles on imaging of microbial cells, because the darkness of an image contrast is simply proportional to average atomic numbers of the atoms in an imaging object (Buseck, 2018). From TEM observations, it was clarified that the dark contrast is derived from the presence of uraninite nanoparticles, with transparent contrast from cellular materials and resin. In addition, cell shapes of *G. sulfureducens* and *D. desulfuricans* were short and curved rods, respectively (Figures 3B,D). The small spheres associated with cuprite nanoparticles in the chimney sample were very similar to microbial cells associated with extracellular uraninite precipitation (Figure 3B; Suzuki et al., 2002, 2003). The image of the small spheres observed in the 150-nm thick FIB section had some contrast (Figures 2E, 3A). This is explained by the effect of small cell size. In sections with a thickness of 150 nm, transparent contrast is expected for cells with large size, regardless of the cell shape (Figure 3G). However, small coccoid cells are visualized with slightly dark contrast (Figure 3G). The image contrast of the small spheres with extracellular Cu_2_O nanoparticles is therefore inferred to be derived from coccoid cells with a small size range. Small greenish spots visualized after DNA staining and the overlapped enrichment of P and CN revealed by NanoSIMS analysis support the conclusion that the small spheres are microbial cells.

Cuprite nanoparticles, which were extracellularly associated with the small spheres (Figures 2F–H), are thermodynamically stable in anoxic and neutral-to-alkaline conditions (Rose, 1976). In the presence of O_2_, the oxidation of cuprite nanoparticles is fast (Lin et al., 2012). These characteristics of cuprite exclude the possibility that the small spheres were contaminants from ambient oxygenated seawater during sampling. The circularity and narrow size distribution of the spheres in the present work supports their biogenicity (Rouillard et al., 2021).

### *In situ* Biosignature Analyses

**FIGURE 4:**
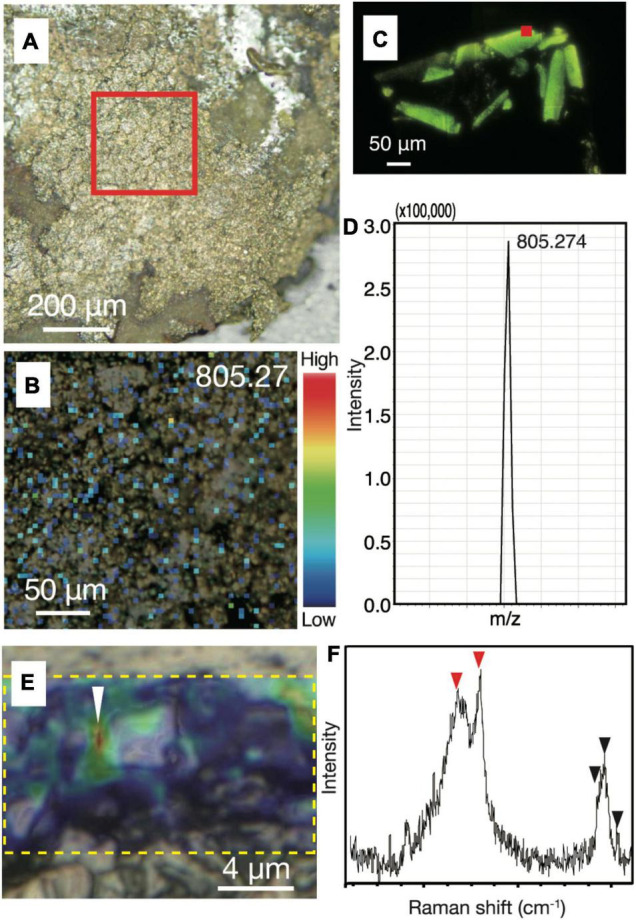
Biological signatures determined by imaging mass spectrometry and Raman spectroscopy from chalcopyrite grain boundaries. **(A)** Reflection light microscope image of inner chalcopyrite wall. The red square highlights the region observed at a higher magnification in Figure 3B. **(B)** Mapping image of a macromolecule at with *m/z* = 805.27 by imaging mass spectrometry analysis with an intensity color contour. **(C)** Fluorescence microscopy image of a DNA-stained section from cultured cells of an archaeon (*Thermococcus kodakarensis*). The red square indicates a spot where a mass spectrum was obtained. **(D)** Mass spectrum focusing on a peak at *m/z* = 805.274 obtained from the spot indicated by the red square in Figure 3C. **(E)** Optical microscope image overlapped with a Raman spectroscopy mapping image of the peak intensity at 2,800–3,000 cm^– 1^, as highlighted by a yellow dotted rectangle. **(F)** Raman spectrum obtained from the region indicated by a white arrow in Figure 3E. Red arrows indicate peaks not found in the resin or minerals spatially associated with the small spheres, while back arrows indicate peaks also found in the resin (Supplementary Figures 5, 6).

To strengthen the evidence that the small spheres are indeed microbial cells, we performed *in situ* biosignature analyses. The spatial distributions of biomolecules such as proteins and lipids in chalcopyrite grain boundaries were characterized by an imaging mass spectrometry using a thin section after SYBR-Green I staining and observations by fluorescence microscopy. Spot analysis of grain boundaries revealed the presence of a macromolecule with *m/z* = 805.27 (Supplementary Figures 5, 6). The mass spectrum of resin was found to lack this macromolecule. Mapping of the macromolecule in the chimney inner wall confirmed its ubiquitous distribution in the grain boundaries (Figures 4A,B). Although the concentration of the macromolecule was not high enough to obtain mass spectra for molecular identification, the presence and absence of the macromolecule in resin and cultured microbial cells, respectively, indicates a biological origin of the macromolecule (Figures 4C,D).

To obtain another line of convincing evidence for the biogenicity of the small spheres (Li et al., 2020), Raman spectroscopy was used to characterize the same region where NanoSIMS analysis was performed on the ribbon-shaped grain boundary (Figure 2C). High-resolution mapping was performed for the peak intensity at 2,800–3,000 cm^–1^, which is attributed to CH_2–3_ typically found in microbial lipids (Figure 4E; Balan et al., 2019). The peak intensity was strong along grain boundaries. Raman spectra obtained from the grain boundaries with the high CH_2–3_ signal were different from those of the resin and minerals spatially associated with the small spheres (Figure 4F and Supplementary Figure 7). The main spectral difference is explained by the presence of CH_3_ and amide groups (C–N and N–H; indicated by red arrows), which is consistent with the distribution of ^12^C^14^N determined by NanoSIMS analysis (Figure 2C).

We considered the possibility that the small spheres are not microbial cells. One possibility is that the spheres are abiotic particulate graphite, as recently discovered in hot and cold vent fluids (Estes et al., 2019). However, the coexistence of C and N and the presence of amide groups in the grain boundaries exclude this possibility. It is known that abiotic carbonaceous matter is formed by rock-water interactions in the oceanic crust (Sforna et al., 2018). In addition, micelles are formed by the self-assembly of lipid bilayers (Monnard and Deamer, 2002; Tang et al., 2014). Even if the small spheres could be produced by abiotic processes, it should be noted that these abiotic products have not been previously observed in the chimney interior.

### Microbial Composition in the Extinct Chimney

To constrain the biogenicity of the small spheres in the chalcopyrite inner wall, the interior and exterior of the extinct chimney was carefully separated for 16S rRNA gene amplicon analysis. Phylogenetic analysis of 16S rRNA gene sequences revealed that members of Pacearchaeota formerly referred to as Deep Sea Hydrothermal Vent Euryarchaeota Group 6 (DHVE-6) were predominant in the chimney interior, remarkably different from the chimney exterior, which was dominated by members of the bacterial phylum Nitrospirae (Supplementary Figure 8 and Supplementary Table 1). It is likely that the remarkable difference in microbial composition can be attributed to textural features of the chimney interior (massive) and exterior (porous) (Figure 1D). This notion is supported by very minor occurrences of Pacearchaeota in previous studies of porous parts of extinct chimneys mainly containing chalcopyrite (Suzuki et al., 2004a; Kato et al., 2010).

By genome-resolved metagenomic analysis, the occurrence and metabolic features of Pacearchaeota have been reported from metal sulfide deposits at deep-sea hydrothermal vents (Kato et al., 2018). The sizes of Pacearchaeota cells in deep sourced groundwater have been measured by flow cytometry coupled to single cell genomics to be ∼200 nm in diameter. The ∼200-nm sized cells densely observed by TEM (Figure 2E) are consistent with the dominance of Pacearchaeota in the chimney interior including chalcopyrite grain boundaries. To identify the ultra-small cells to be Pacearchaeota, fluorescence *in situ* hybridization was performed with probes targeting archaea and Pacearchaeota for thin sections (Supplementary Figure 9; Nussbaumer et al., 2006). As cells were not hybridized with the probes at chalcopyrite grain boundaries, we could not phylogenetically determine the ultra-small cells. However, the dominant occurrence of Pacearchaeota supports the presence of the ultra-small cells in the chimney interior.

## Discussion

### Factors Controlling Microbial Distribution in Chalcopyrite Grain Boundaries

Nanosolid and biosignature analyses revealed microbial cells associated with the mineral assemblages inside the extinct chimney. Our investigations are specific at the grain boundary, as opposed to the bulk analysis of the chimney interior after physical separation (Suzuki et al., 2004a; Kato et al., 2010; Sylvan et al., 2012, 2013). This is the first evidence of microbial cells densely occurring in the grain boundaries of a deep-sea hydrothermal vent chimney. We, hereafter, consider geochemical and microbial processes involved in the formation of the chalcopyrite grain boundaries densely packed with microbial cells. Fluid temperatures of vent chimneys are above 300°C (Nakamura et al., 2013). At this initial stage of chimney formation, no cellular forms containing nucleic acids are present, because nucleic acids are thermally unstable above 150°C (Leibrock et al., 1995). Hence, microbial cells appear in the inner chimney wall after waning of hydrothermal activity from high- to low-temperature fluid venting.

We postulated two possibilities for the timing of distribution of the microbial cells in the grain boundaries of the inner chimney. One is entrapment of subvent microorganisms transported in low-temperature fluids. This possibility is supported by metabolically diverse microbes in low-temperature fluids (Reysenbach et al., 2020). The other possibility is the proliferation of microorganisms in the chimney grain boundaries after its cessation; if this is the case, the microorganisms would be energetically dependent on chemicals dissolved from chalcopyrite in the extinct chimney (Suzuki et al., 2004a; Kato et al., 2010), despite the slow dissolution rate of chalcopyrite in anoxic and pH-neutral conditions (Kimball et al., 2010).

Although we were unable to determine whether the microbial cells are alive or growing in the extinct chimney, chalcopyrite grain boundaries serving as space for dense microbial cells in the chimney interior are a new environment in the deep-sea hydrothermal vent ecosystem. Our findings potentially expand the photosynthesis-independent ecosystem where grain boundaries in rock matrix are isolated from organics and O_2_ produced by photosynthesis. The existence of submarine chalcopyrite deposits is dated back to 3.25 billion years ago (Rasmussen, 2000), suggesting that such rocky habitants play important roles in survival and diversification of ultra-small cells (Reysenbach et al., 2020). In future studies, metabolic activities in chimney grain boundaries need to be determined by *in situ* stable isotope labeling coupled with the nanosolid and biosignature analyses developed in this study (Wagner, 2009).

## Materials and Methods

### Sample Collection and Subsampling

A metal sulfide chimney was collected from an active hydrothermal vent field at the Pika site (12°55.15′N, 143°36.96′E) in the Southern Mariana Trough during the Japan Agency for Marine-Earth Science and Technology (JAMSTEC) Scientific Cruises NT12-24 of the R/V *Natsushima* in September of 2012. The Southern Mariana Trough is a back-arc basin where the Philippine Sea Plate is subducted (Figure 1A). The chimney structure visually confirmed for the lack of hydrothermal fluid venting was collected by the manipulator arm of remotely operated vehicle (ROV) *Hyper Dolphin* (Figure 1B). The metal sulfide chimney was placed into an enclosed container to minimize contamination from surrounding seawater during transportation to the surface.

The collected chimney was immediately subsampled onboard in a cold room at 4°C. Using sterile chisels and spatulas, the exterior portion of the sample was subsampled. For subsampling of the interior portion, the chimney surface was flamed by a gas torch to prevent contamination from the exterior potion. Some intact portions of the chimney structure were preserved for light and electron microscopic observations, μ-Raman spectroscopy, imaging mass spectrometry, and nanoscale secondary ion mass spectrometry (NanoSIMS) analysis for mineral and microbial distributions, while the rest of the chimney structure was ground into powder by using sterile pestles and mortars. Both the intact and the ground subsamples were fixed with 3.7% formamide in seawater onboard. The rest of the subsamples were frozen at −80°C for DNA extraction and mineralogical characterizations. All solutions were filtered with a 0.22-μm-pore-size filter (Millipore).

### Mineralogical and Microbiological Characterizations of Thin Sections From the Metal Sulfide Chimney

To clarify mineral composition and microbial distribution within the chimney interior, thin sections were prepared using LR White resin. The intact chimney subsamples were dehydrated twice in 100% ethanol for 5 min, and then infiltrated four times with LR White resin (London Resin Co. Ltd.) for 30 min and solidified in an oven at 50°C for 48 h. Solidified blocks were trimmed into thin sections and polished with corundum powder and diamond paste. For the staining of microbial cells embedded in LR White resin, TE buffer with SYBR Green I (TaKaRa-Bio, Inc.) was mounted on thin sections and covered with cover glasses. After dark incubation for 5 min, thin sections rinsed with deionized water and mounted with the antifade reagent VECTASHIELD (Vector Laboratories) were observed using a fluorescence microscope (Olympus BX51) and a charge-coupled device (CCD) camera (Olympus DP71). Two ranges of fluorescence between 540–570 and 570–600 nm were used to discriminate microbial cells from mineral-specific fluorescence signals.

Mineral assemblages and textures were observed using the same microscope with the transmission light mode. Reflection light microscopy observations were conducted by an optical microscope (Nikon ECLIPSE E600POL E6TP-M61) and a CCD camera (Zeiss AxioCam MRc 5). Carbon-coated thin sections were characterized using a scanning electron microscope (Hitachi S4500) at an accelerating voltage of 15 kV. Back-scattered electron imaging coupled to EDS was used to analyze the chemical compositions of mineral phases according to image contrasts.

To analyze microbial cells found in thin sections by NanoSIMS at the Kochi Institute for Core Sample Research (KOCHI), JAMSTEC (CAMECA NanoSIMS 50L), 3-μm, 300-nm, and 150-nm thick sections were fabricated using FIB sample-preparation and micro-sampling techniques using a Hitachi FB-2100 instrument at the University of Tokyo or a Hitachi SMI-4050 at KOCHI. The thin-section samples were locally coated with the deposition of W (100–500-nm thick) for protection and trimmed using a Ga-ion beam at an accelerating voltage of 30 kV. A focused primary Cs^+^ ion beam of approximately 1.0 pA (100-nm beam diameter) was rastered on the samples. Secondary ions of ^12^C^–^, ^16^O^–^, ^12^C_2_^–^, ^12^C^14^N^–^, ^28^Si^–^, ^31^P^–^, and ^32^S^–^ were acquired simultaneously with multidetection using seven electron multipliers at a mass-resolving power of approximately 4,500. Each run was initiated after stabilization of the secondary ion-beam intensity following presputtering of <approximately 2 min with a relatively strong primary ion-beam current (approximately 20 pA). Each imaging run was repeatedly scanned (15–20 times) over the same area, with individual images comprising 256 × 256 pixels. The dwell times were 5,000 μs/pixel for the measurements, and total acquisition time was approximately 2 h. The images were processed using the NASA JSC imaging software for NanoSIMS developed by the Interactive Data Language program (Ito and Messenger, 2008).

Transmission electron microscopy (TEM) was used to examine microbial distributions and the structure and composition of minerals at the nanometer scale. JEOL JEM-2010 with EDS was operated at 200 kV at the University of Tokyo. A JEOL JEM-ARM200F transmission electron microscope was used at an accelerating voltage of 200 kV at KOCHI, JAMSTEC.

### Imaging Mass Spectrometry

An atmospheric pressure matrix-assisted laser desorption/ionization system equipped with a quadrupole ion trap-time of flight analyzer (MALDI-TOF) was used to obtain MS data from spots or regions observed by microscopy (iMScope *TRIO*, Shimadzu). The thin section subjected to nanosolid characterizations was further thinned by a mechanical polisher (Leica EM TXP Target Preparation Device). The thin section was coated with 9-aminoacridine (Merck) by iMLayer (Shimadzu) with a thickness of 1.0 μm and then irradiated by a 355 nm Nd:YAG laser with a laser diameter of ∼5 μm. The positive ion mode was used for imaging of the thin section. Scanning was performed with a pitch of 5 μm. Operation conditions were as follows: frequency, 1,000 Hz; 50 shots per spot. To visualize the ion images, Imaging MS Solution (Shimadzu) was used. For a reference, LR White resin sectioned to a thickness of 20 μm using an ultramicrotome was mounted on the carbon tape. As another reference, cultured cells of *Thermococcus kodakarensis* (JCM 12380) were embedded in LR White resin sectioned to a thickness of 12.5 μm and mounted on a carbon tape.

### Raman Spectroscopy

A high-resolution confocal Raman system (Horiba LabRam HR) equipped with a laser (532 nm) was used to characterize chalcopyrite grain boundaries on a thin section. The incident laser was operated at 1.2–4 mW. Analytical uncertainty in the Raman shift was ∼2 cm^–1^, and the spatial resolution was ∼1 μm. Raman spectra were compared with standard spectra obtained from RRUFF,^1^ and peak assignments were based on references (Schneider et al., 1979; Sodo et al., 2016; Qian et al., 2020).

### Uranium Reduction Experiments and Transmission Electron Microscopy Observations of Incubated Cells

*Desulfovibrio desulfuricans* [ATCC#642] and *Geobacter sulfurreducens* [ATCC#51573] were subjected to uranium reduction experiments previously performed for *Desulfosporosinus* spp. (Suzuki et al., 2004b). In an anaerobic glove box with a gas mixture of N_2_-CO_2_-H_2_ (90:5:5), cells grown in media recommended by ATCC were incubated in 0.25% bicarbonate solution at pH 7 containing 1 mM of uranyl acetate. A mixture of 10 mM sodium lactate and 10 mM sodium acetate was added for electron donors of *D. desulfuricans* and *G. sulfurreducens*, respectively. After 24-h incubation, cells were harvested by centrifugation at 10,000 × *g* for 3 min. Whole mounts of the incubated cells of *D. desulfuricans* and *G. sulfurreducens* were observed by TEM (JEOL JEM-2010). The incubated cells of *D. desulfuricans* were embedded in LR White resin as described above, and 100-nm thick sections prepared with an ultramicrotome (Ultracut S, Reichert-Nissei, Tokyo, Japan) were also observed by TEM.

### DNA Extraction and 16S rRNA Gene Amplicon Analysis

Prokaryotic DNA was extracted from 0.1 g of frozen powdered chimney subsamples as described previously (Kouduka et al., 2012). In brief, the powdered chimney subsample was incubated at 65°C for 30 min in 300 μl of alkaline solution consisting of 75 μl of 0.5 N NaOH and 75 μl of TE buffer (Nippon Gene Co.) including 10 mM Tris–HCl and 1 mM EDTA. After incubation, the aliquots were centrifuged at 5,000 × *g* for 30 s. Then, the supernatant was transferred into a new tube and neutralized with 150 μl of 1 M Tris–HCl (pH 6.5; Nippon Gene Co.). After neutralization, the DNA-bearing solution (pH 7.0–7.5) was concentrated using a cold ethanol precipitation, and the DNA pellet was dissolved in 50 μl of TE buffer. The purified DNA solution was stored at −4 or −20°C for a longer time. For the negative control, DNA extraction from the subsample was performed in parallel with one extraction negative control to which no sample was added.

The 16S rRNA gene was amplified using LA Taq polymerase (TaKaRa-Bio, Inc.). For pyrosequencing, the 454 GS-junior sequencer (Roche Applied Science) was used. The primers Uni530F and Uni907R (Nunoura et al., 2009) were extended with adaptor sequences (CCATCTCATCCCTGCGTGTCTCCGACTCAG for Uni530F and CCTATCCCCTGTGTGCCTTGGCAGTCTCAG for Uni907). The forward primer Uni530 was barcoded with 8-mer oligonucleotides (Hamady et al., 2008). Thermal cycling was performed with 35 cycles of denaturation at 95°C for 30 s, annealing at 58°C for 45 s, and extension at 72°C for 1 min. A PCR product with the expected size was excised from 1.5% agarose gels after electrophoresis on TAE (40 mm Tris acetate, 1 mm EDTA, pH 8.3), which was purified using MinElute Gel Extraction Kit (Qiagen, Inc.). A DNA concentration of the purified PCR product was measured by the Quant-iT dsDNA HS assay kit and the Qubit fluorometer (Invitrogen, Inc.). The concentration of total double-stranded DNA in each sample was adjusted to 5 ng/μl. Emulsion PCR was performed using the GS FLX Titanium emPCR Kit Lib-L (Roche Applied Science) to enrich DNA library beads for the 454 GS-junior sequencers. Amplified DNA fragments were sequenced according to the manufacturer’s instructions.

Raw reads were demultiplexed, trimmed, and filtered based on their 8-bp sample-specific tag sequences, quality values (QVs), and lengths using Mothur v. 1. 31 (Schloss et al., 2009) to obtain unique reads more than 250 base pairs (bp), and an average quality score>27. Filtered sequences were aligned with Mothur to the Greengenes reference database (DeSantis et al., 2006), and chimeric sequence reads were removed with Chimera Uchime in Mothur. Sequence reads were clustered into operational taxonomic units (OTUs) sharing 97% identity within each OTU. Phylogenetic affiliations of the OTUs were assigned by the neighbor joining method in the ARB software package, along with closely related sequences retrieved from GenBank^2^ through BLASTn searches (somewhat similar sequences). The 16S rRNA gene sequences in this study were all deposited in the DDBJ nucleotide sequence database with accession numbers LC554901-LC555740.

### Fluorescence *in situ* Hybridization Analysis

Whole-cell hybridization was performed for thin sections of the intact chimney subsample embedded in LR-White resin. Hybridization was conducted at 46°C in a solution containing 20 mM Tris–HCl (pH 7.4), 0.9 M NaCl, 0.1% sodium dodecyl sulfate, 30% (v/v) formamide, and 50 ng/μl of each probe labeled at the 5′ end with fluorescence dye. A Cy-5 labeled probe targeting the domain Archaea (Arch915: 5′-GTGCTCCCCCGCCAATTCCT-3′) (Alm et al., 1996) and a Cy-5 labeled probe targeting Patharchatta (Pace915: 5′-GTGTCTCCCCGCCAATTCCT-3′) were used for hybridization of the positive control and the chimney thin sections, respectively. After hybridization, the specimens were washed at 48°C in a solution lacking the probes and formamide at the same stringency, adjusted by NaCl concentration. After staining with 4′,6-diamidino-2-phenylindole (DAPI) at 0.4 μg/ml, the slides were examined using the Olympus BX51 microscope. For the positive control, cultured archaeal cells of *Methanocaldococcus* sp. Mc-365-70 were used. For the negative control, a bacteria-specific probe named EUB338 (5′-GCT GCC TCC CGT AGG AGT-3′) (Amann et al., 1990) was used to check the absence of non-specific binding of the EUB338 probe on the cultured archaeal cells under the same hybridization conditions.

## Data Availability Statement

The original contributions presented in this study are included in the article/Supplementary Material, further inquiries can be directed to the corresponding author.

## Author Contributions

HT and YS designed the study and conducted Raman spectroscopy. YS, HF, and SK collected and analyzed the chimney sample as shipboard scientists during JAMSTEC Scientific Cruises NT12-24. HT, HM, YK, NT, and YS performed mineralogical characterizations. HT, MI, and YS conducted NanoSIMS analysis. HT, TY, and YS performed MALDI-TOF-MS analysis. YS performed uranium reduction experiments. HT, MK, and YS co-wrote the manuscript. All authors discussed the results and commented on the manuscript.

## Conflict of Interest

KN and TY were employed by Shimadzu Corporation. YK was employed by TOYO Corporation. The remaining authors declare that the research was conducted in the absence of any commercial or financial relationships that could be construed as a potential conflict of interest.

## Publisher’s Note

All claims expressed in this article are solely those of the authors and do not necessarily represent those of their affiliated organizations, or those of the publisher, the editors and the reviewers. Any product that may be evaluated in this article, or claim that may be made by its manufacturer, is not guaranteed or endorsed by the publisher.

## Footnotes

1
http://rruff.info

2
http://www.ncbi.nlm.nih.gov/genbank/
