# Supplementary material for: Copper-Nanocoated Ultra-Small Cells in Grain Boundaries Inside an Extinct Vent Chimney
Source: Front Microbiol. 2022 Jun 7;13:864205. doi: 10.3389/fmicb.2022.864205 (PMC9209642; doi:10.3389/fmicb.2022.864205)
Supplement: Supplementary file 1 [file Data_Sheet_1.docx]

Supplementary Material

# Copper-nanocoated ultra-small cells in grain boundaries inside an extinct vent chimney

# Hinako Takamiya^1^, Mariko Kouduka^1^, Hitoshi Furutani^1^, Hiroki Mukai^2^, Kaoru Nakagawa^3^, Takushi Yamamoto^3^, Shingo Kato^4^, Yu Kodama^5^, Naotaka Tomioka^6^, Motoo Ito^6^, and Yohey Suzuki^1^

# ^1^Department of Earth and Planetary Science, The University of Tokyo, 7-3-1 Hongo, Bunkyo-ku, Tokyo 113-0033, Japan

# ^2^Faculty of Life and Environmental Sciences, University of Tsukuba, Tennodai 1-1-1, Tsukuba, Ibaraki 305-8572, Japan

^3^ Solutions COE, Analytical & Measuring Instruments Division, Shimadzu Corporation, 1, Nishinokyo Kuwabara-cho, Nakagyo-ku, Kyoto 604-8511, Japan

# ^4^Japan Collection of Microorganisms (JCM), RIKEN BioResource Research Center, 3-1-1 Koyadai, Tsukuba, Ibaraki 305-0074, Japan ^5^TOYO Corporation,^5^TOYO Corporation, 1-6, Yaesu 1-chome, Chuo-ku, Tokyo 103-8284, Japan ^6^Kochi Institute for Core Sample Research, Japan Agency for Marine-Earth Science and Technology (JAMSTEC), Monobe B200, Nankoku, Kochi 783-8502, Japan

# *Yohey Suzuki

# Email: yohey-suzuki@eps.s.u-tokyo.ac.jp

# This file includes:

# Figures S1 to S9

#
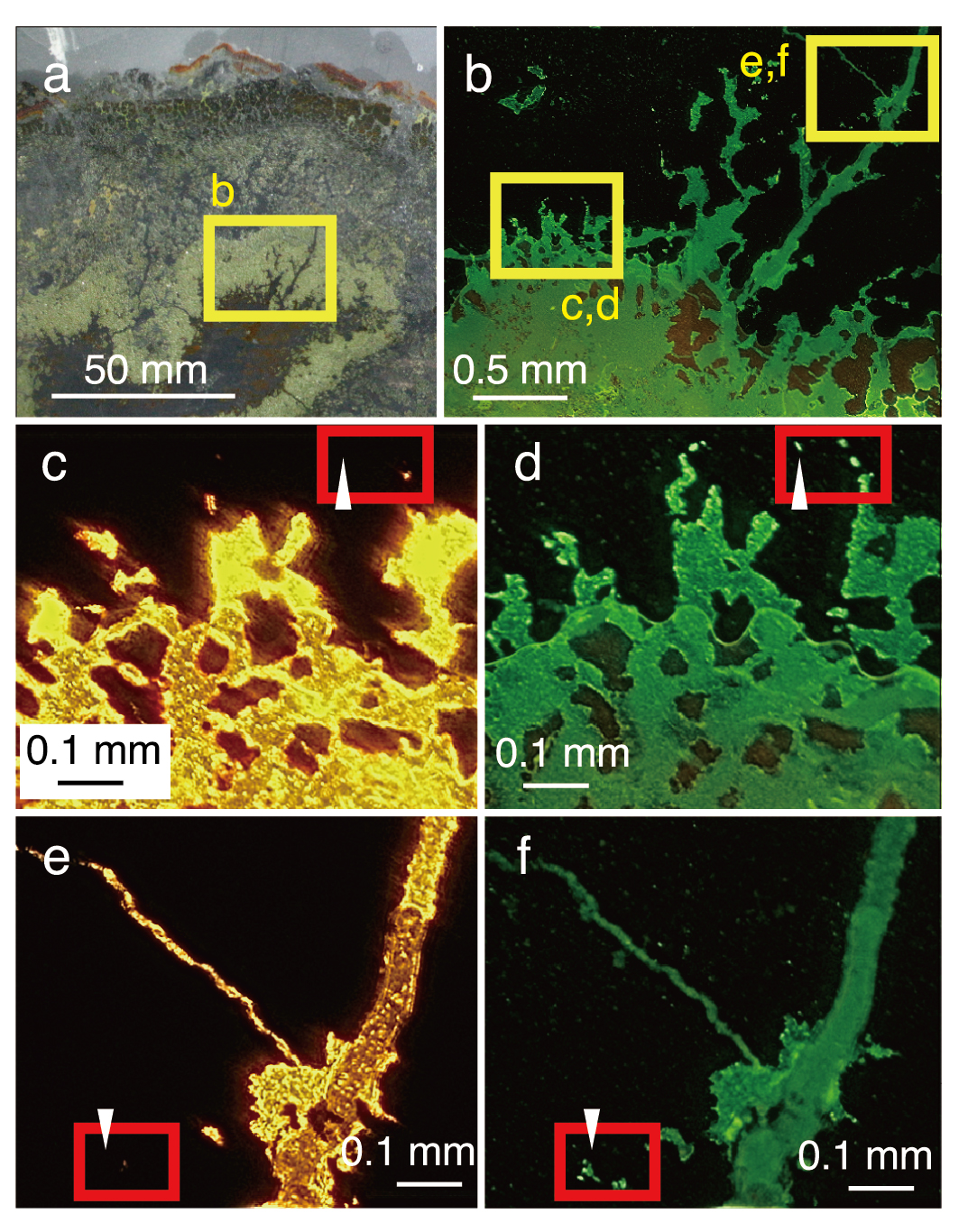


# Supplementary Figure 1. Light and fluorescence microscopy images of a SYBR Green I-stained thin section from the extinct chimney. a, Optical microscope image. b, Low-magnification fluorescence image. c, Middle-magnification transmitted-light image. d, Middle-magnification fluorescence image. e, Middle-magnification transmitted-light image. f, Middle-magnification fluorescence image. Yellow rectangles highlight areas observed at higher magnifications in separate images. Red rectangles and arrows highlight SYBR-Green stained grain boundaries without light transmission.

#
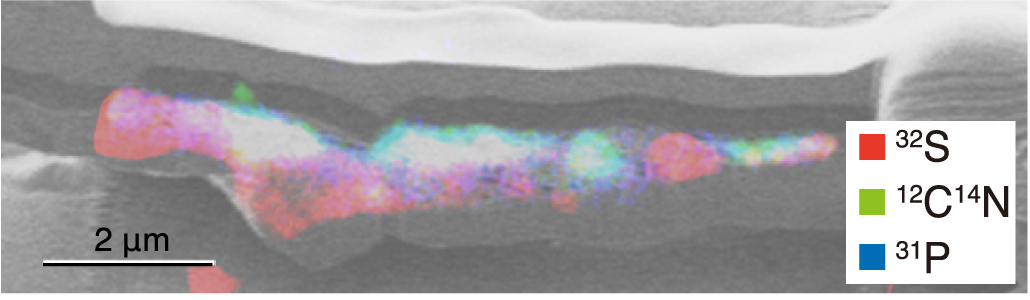


# Supplementary Figure 2. Elemental distribution in a silicate-bearing layer with sub-micron voids. Overlay image of a Ga ion image in black and white and the Nanoscale secondary ion mass spectrometry (NanoSIMS) images of ^32^S in red, ^12^C^14^N in green, and ^31^P in blue.

#
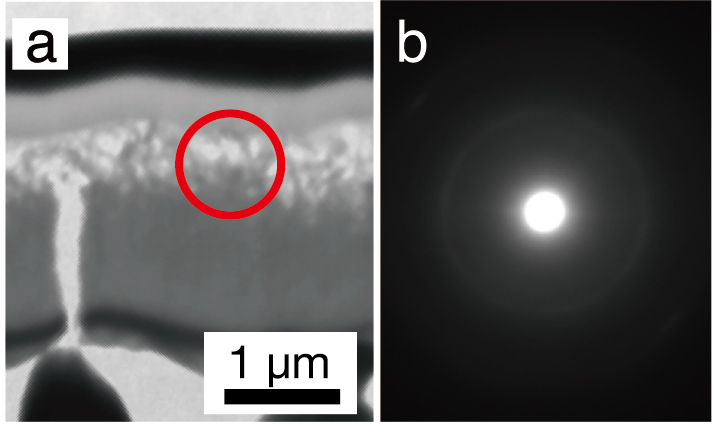


# Supplementary Figure 3. Mineralogical characterizations of a fibrous silica-bearing layer in a 300-nm thick FIB section. a, Transmission electron microscope (TEM) image. b, A selected area electron diffraction (SAED) pattern. Extended Fig. 3b was taken from a red circle in Extended Fig. 3a.

#
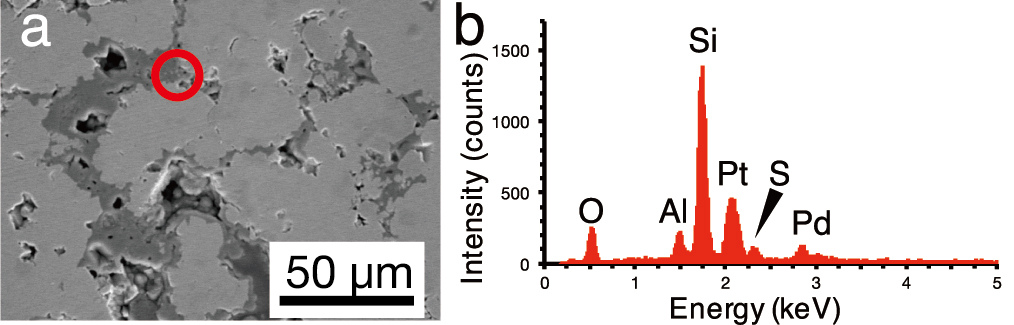


# Supplementary Figure 4. Mineralogical characterizations of a region fabricated for a 150-nm thick FIB section. a, Scanning electron microscope (SEM) image. b, Energy-dispersive X-ray spectroscopy (EDS) spectrum of a red circle in Extended Fig. 4.

#
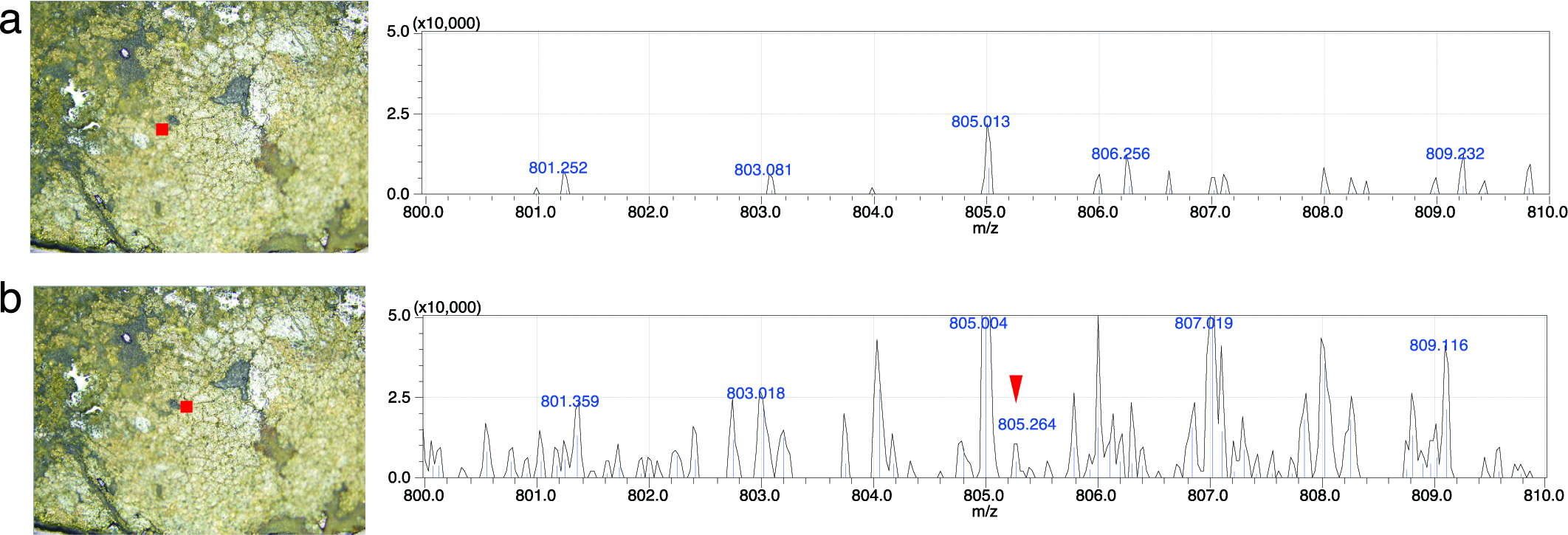


# Supplementary Figure 5. Imaging mass spectrometry analysis of grain boundaries of the inner chalcopyrite wall. Spot analysis was conducted at each filled square in the left images. a-b, Chalcopyrite grain boundary.

#
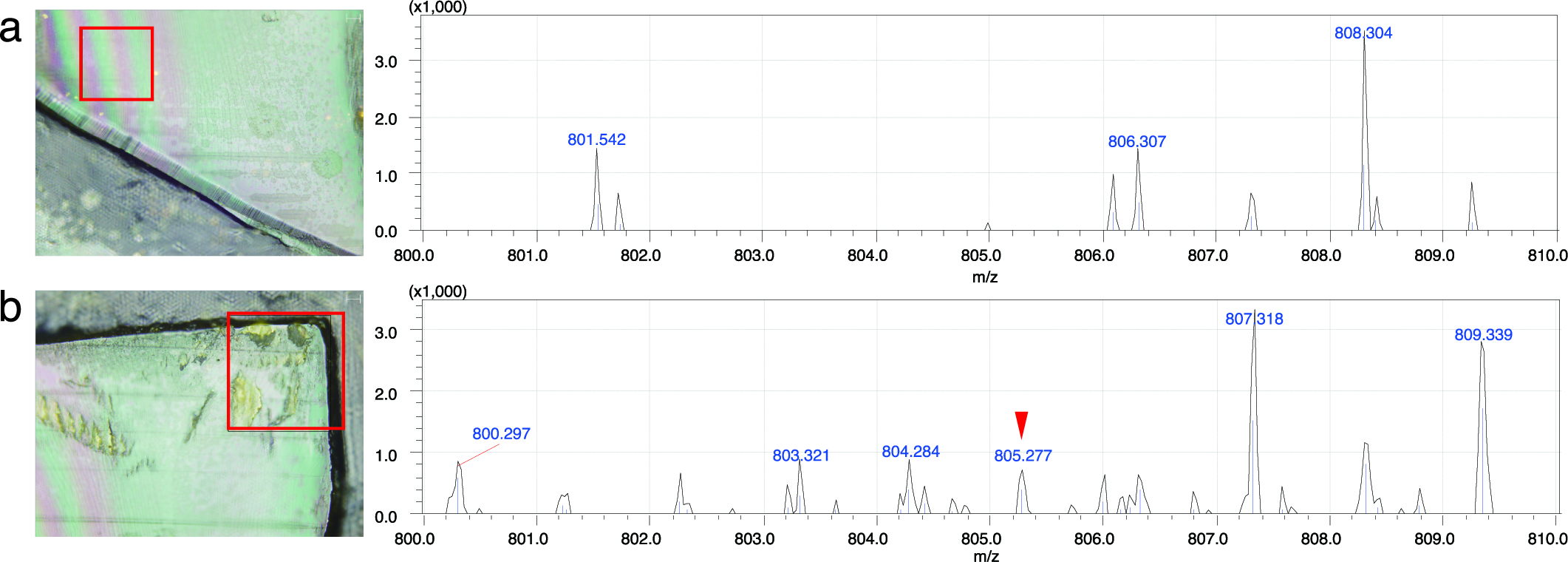


# Supplementary Figure 6. Imaging mass spectrometry analysis of a 12.5-μm thick section of cultured cells of *Thermococcus kodakarensis.* Scan analysis was conducted in each red square. a, resin. b, cell aggregates.

#
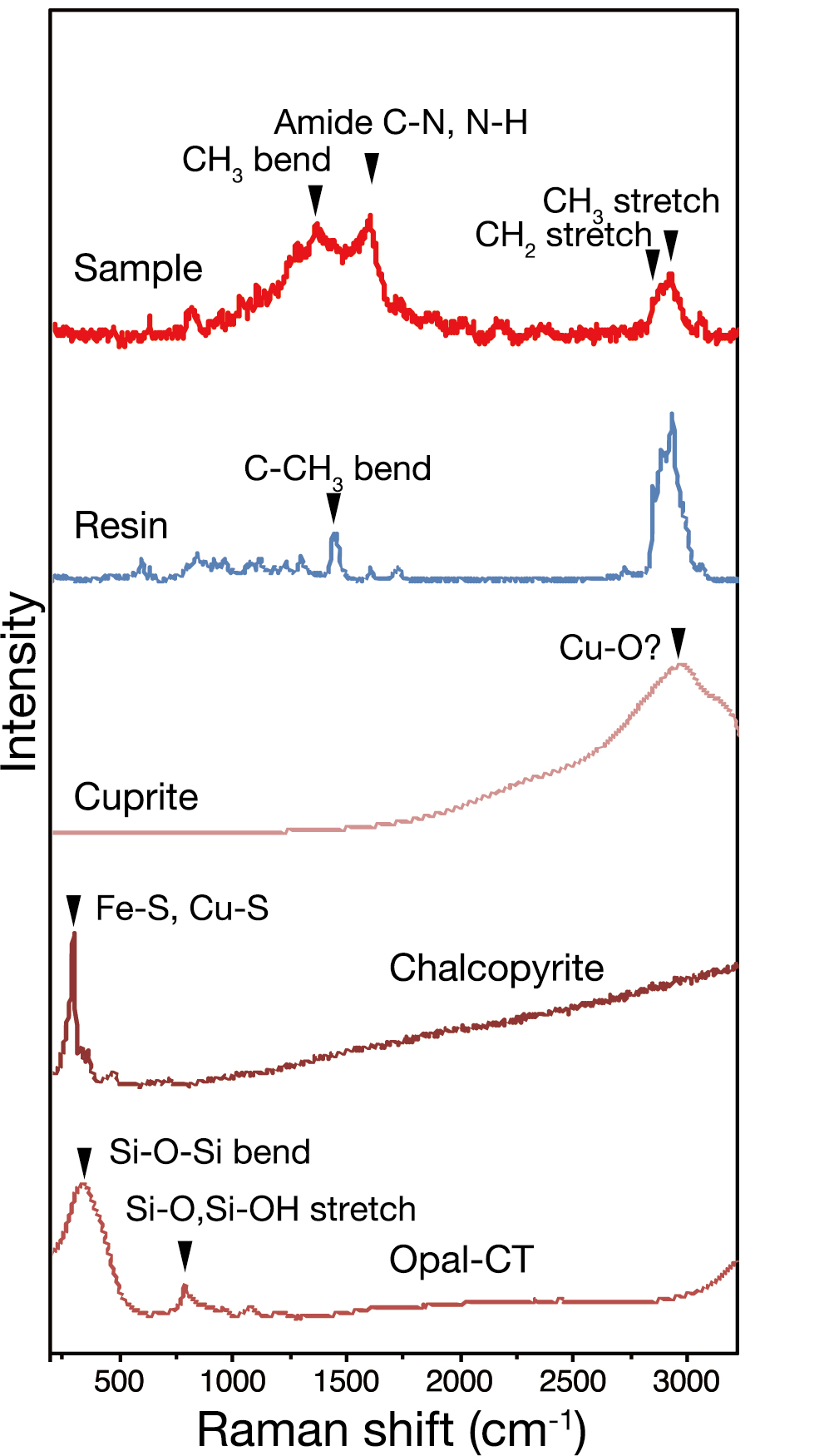


# Supplementary Figure 7. Comparison of Raman spectra from a grain boundary in the inner chalcopyrite wall and references. A spectrum marked with “sample” is also shown in Fig. 4b. A spectrum marked with “resin” was obtained from LR White resin. Cuprite, chalcopyrite and opal-CT were shown as spectra obtained from RRUFF (http://rruff.info)(Lafuente et al. 2015). Peak positions in opal-CT are almost the same as those in amorphous silica (Sodo et al. 2016).


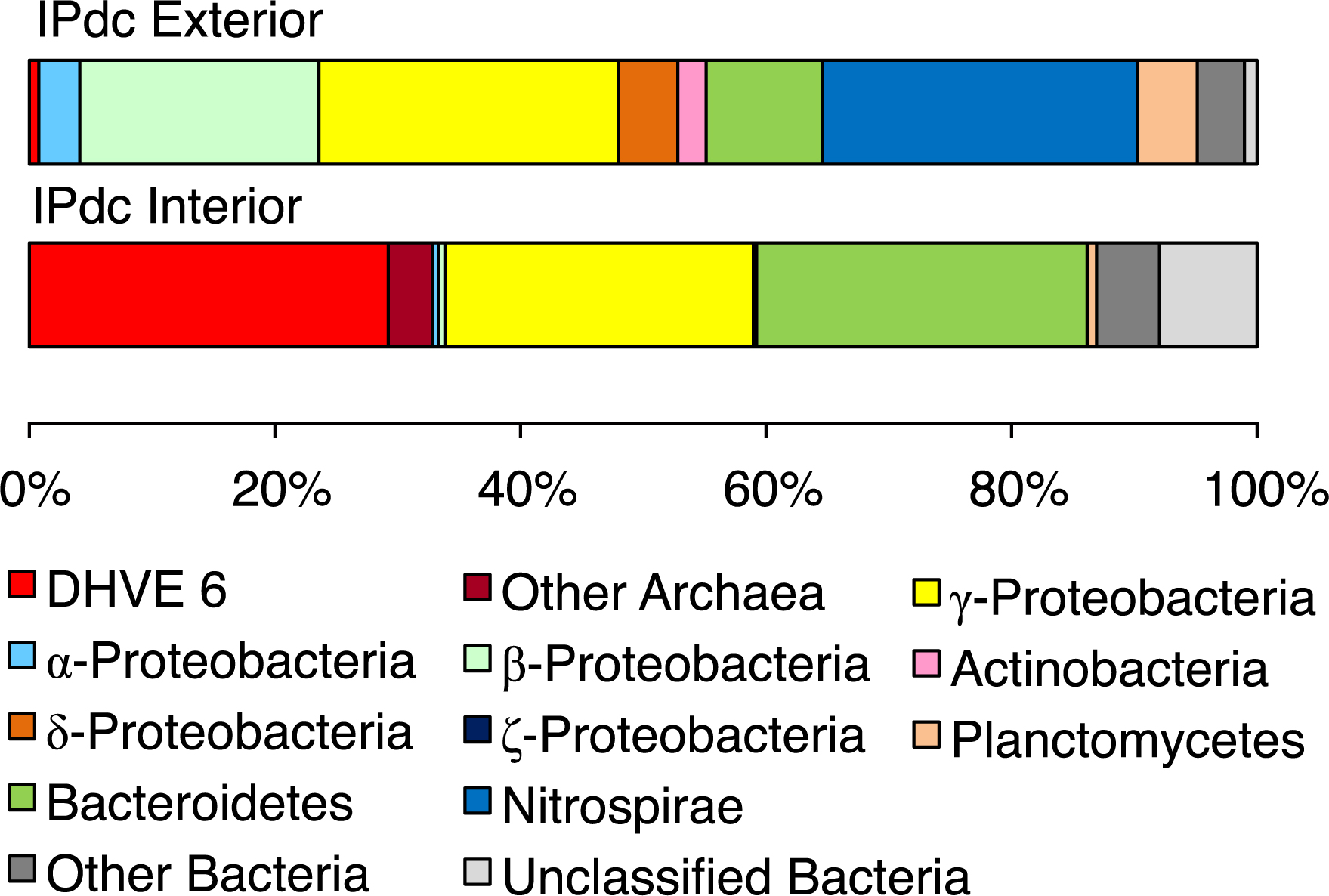


**Supplementary Figure 8. Microbial community structures based on prokaryotic 16S rRNA gene sequences from the chimney interior and exterior.** The relative abundances of sequences classified by phylum or class are shown.


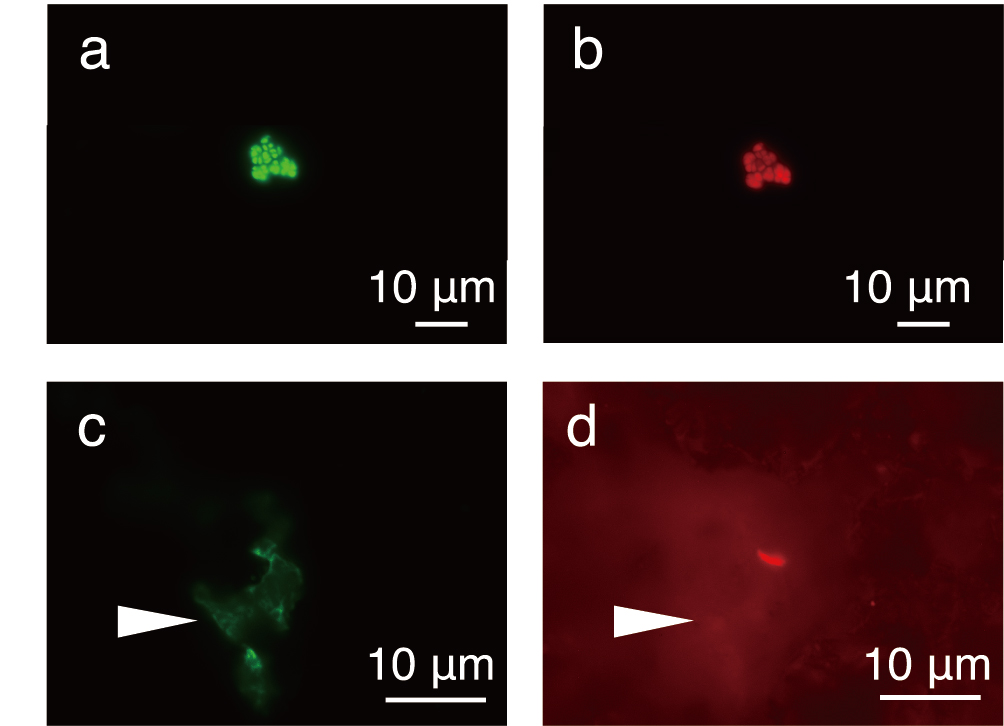


**Supplementary Figure 9. Application of FISH to resin-embedded microbial cells in thin sections.** **a-b**, Cultured cells of *Methanocaldococcus* sp. Mc-365-70 stained with SYBR-Green I (left) and a Cy5-labelled Archaea-universal probe called Arch915 (right). **c-d**, Grain boundary of the inner chalcopyrite wall stained with SYBR-Green I (left) and a Cy5-labelled Pacearchaeota-targeted probe called Pace915 (right). A white arrow indicates a grain boundary region, in which greenish spots stained by SYBR-Green I (left) are not associated with Cy5-derived signals (right).

# References

Lafuente**,** B**.**, Downs**,** R**.**, Yang**,** H**.**, & Stone**,** N**.** (2015) The power of databases: the RRUFF project. In “Highlights in mineralogical crystallography”, Armbruster, T. & Danisi, RM, eds. W. *De Gruyter, Berlin, Germany* 1:30.

Sodo, A., Casanova Municchia, A., Barucca, S., Bellatreccia, F., Della Ventura, G., Butini, F., & Ricci, M. A. (2016) Raman, FT‐IR and XRD investigation of natural opals. *J Raman Spectrosc* 47:444-1451. doi.org/10.1002/jrs.4972
